# Nitrogen Regulation and Its Chemical State in FeCr17Mn12Mo3.5N Powders Based on High-Pressure Nitriding

**DOI:** 10.3390/ma19102053

**Published:** 2026-05-14

**Authors:** Xiaofei Jiao, Yubiao Song, Yanxiao Li, Rui Xie, Shuhuan Wang, Xiangming Che, Qun Li, Guolong Ni

**Affiliations:** 1 School of Metallurgy and Energy, North China University of Science and Technology, Tangshan 063210, China; 13676962634@stu.ncst.edu.cn (X.J.);; 2 Hebei Iron and Steel Laboratory, North China University of Science and Technology, Tangshan 063210, China

**Keywords:** high-nitrogen stainless steel powder, high-pressure metallurgy, ultra-high nitrogen content, nitriding mechanism, nitride regulation

## Abstract

**Highlights:**

1. An innovative process combining high-pressure metallurgy and solid-state powder nitriding is proposed to prepare UHNASS powders.
2. Ultra-high-nitrogen powders with a single γ-austenite phases can be successfully obtained, and their nitrogen content reached 5.63 wt.%.
3. N elements is uniformly distributed in the powder matrix and mainly exists in the form of a solid solution with a small amount of dispersed nanoscale nitrides.
4. The mechanisms of nitrogen increase and nitrogen control under high pressure have been explored in detail.

**Abstract:**

The demand for high-nitrogen austenitic stainless steel (HNASS) powders has become increasingly urgent due to the rapid development of advanced manufacturing processes. However, it still remains a challenge to accurately control the nitrogen content and its chemical state. In this work, an innovative process combining high-pressure metallurgy and solid-state powder nitriding is proposed to prepare ultra-high nitrogen austenitic stainless steel powders. The prepared powders not only exhibit fine austenite grains, but also achieve a high nitrogen content of up to 5.63 wt.% at 1000 °C, 2.5 h, and 2.5 MPa. The results demonstrate that the phase composition of the powders, as well as the size and distribution of nitrides can be effectively regulated by carefully controlling the key processing parameters including temperature, time and pressure. Nitrogen is predominantly uniformly distributed as solid solution, with minor nanoscale nitride precipitates. XPS analysis of the powder surface indicates that the peak area ratios of N1s (N 1s core-level) in the form of solid solution and nitrides are ~82.95% and ~17.05%, respectively. And the peak area ratios of N1s at different depths of the powder do not show significant changes. Furthermore, the high-pressure nitriding mechanism reveals that the synergy between a high-pressure nitrogen atmosphere and solid-state nitriding enhances nitrogen diffusion flux and increases nitride nucleation density, enabling precise control of nitrogen content and precipitate size. Moreover, the high-pressure nitriding process can effectively keep nitrogen in a solid solution, prevent the precipitation of coarse nitrides, and consequently improve the quality of the powders. This research provides in-depth guidance and insights into the design and preparation of HNASS powders.

## 1. Introduction

High-nitrogen austenitic stainless steels (HNASSs), as a new type of engineering material, exhibit excellent wear and corrosion resistance, favorable mechanical properties and ideal biocompatibility, which renders them suitable for wide applications in the petrochemical, aerospace, ocean engineering and biomedical fields [1,2,3]. In recent years, with the rapid development of powder metallurgy and additive manufacturing technology, the demand for HNASS powders has become increasingly urgent. Furthermore, it is well known that the properties of metal powders have a significant effect on the final HNASS components [4,5,6]. Thus, it is a crucial aspect of efforts to obtain high-quality HNASS powders for additive manufacturing technology.

Nitrogen acts as a key element in forming and stabilizing austenite. Through solid-solution strengthening, it greatly enhances the mechanical performance and corrosion resistance of HNASSs [7,8]. Meanwhile, nitrogen can partially replace nickel. This substitution cuts material costs and prevents nickel allergy risks [9,10]. Previous studies have generally reported nitrogen contents ranging from 0.4 wt.% to 0.9 wt.% which is related to the composition and preparation process of the steel [11,12,13,14]. In addition, it is well known that the distribution and chemical state (i.e., solid solution nitrogen or nitrides) of nitrogen strongly affect the comprehensive properties of HNASSs. For example, alloying elements such as Cr, Ti, and Nb have a strong affinity for nitrogen, which promotes the formation of stable nitrides [15,16]. Therefore, it is necessary to avoid the excessive and coarse formation of nitrides, as such a situation would seriously deteriorate the mechanical properties of HNASSs [17,18]. Furthermore, the formation of coarse nitrogen-chromium compounds immobilizes chromium atoms within the precipitates, rendering them unavailable to form the protective passive film and thus deteriorating the corrosion resistance of the stainless steel [19,20]. Fortunately, the above problems can be effectively mitigated by regulating the size and distribution of nitrides [21,22,23], which is closely related to the distribution and existence status of nitrogen in HNASSs. Therefore, it is highly meaningful and valuable to explore an appropriate method for achieving nitrogen control. Various synthetic methods have been adopted to prepare HANSS powders, such as plasma rotating electrode atomization, mechanical alloying, gas atomization and solid powder nitriding [24,25,26]. Among them, the solid powder nitriding method has attracted considerable attention owing to the advantage of achieving an efficient synergy between “nitrogen increasing” and “nitrogen controlling”. Meanwhile, this process can effectively meet the high-quality requirements of powder raw materials for advanced manufacturing technology [27,28]. Nevertheless, it still suffers from excessively long production cycles, low efficiency, poor nitrogen control capability, and a tendency for powder cracking [29,30,31]. Moreover, studies on high-pressure powder nitriding remain very limited. In particular, the synergistic effects of a high-pressure nitrogen atmosphere and solid-state powder nitriding on nitrogen diffusion flux, nitride nucleation density, and the precise control of nitrogen content and chemical state have not been systematically investigated [32,33,34].

To address the above problems, an innovative process is proposed in this work by combining the solid powder nitriding process with high-pressure metallurgy to achieve precise control of “nitrogen increasing” and “nitrogen controlling”. A high-pressure nitrogen atmosphere is introduced into the process of solid powder nitriding to enhance the nitriding kinetics of HNASS powders, and further achieve the efficient and controllable preparation of ultra-high nitrogen austenitic stainless steel powders. The characterization of the as-prepared powders is executed using multiple techniques. A systematic investigation is conducted to elucidate the effects of key processing parameters, namely nitriding pressure, temperature and time, on the nitrogen content, phase composition, and microstructure of the as-prepared powders. In addition, the mechanism of nitrogen control is explored, which will provide guidance and insights for the controllable preparation of high-quality HNASS powders.

## 2. Materials and Methods

### 2.1. Material Preparation

**Figure 1:**
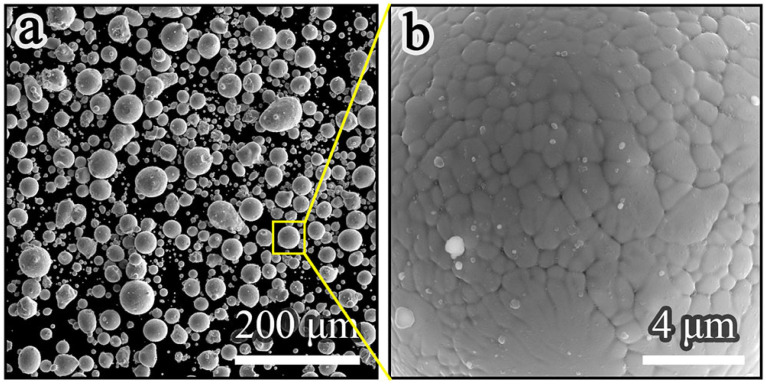
SEM images of raw powders: (**a**) external morphology; (**b**) locally external morphology.

The raw material used in this study was gas-atomized FeCr17Mn12Mo3.5N powder with a particle size of 15–53 μm. The powder was a duplex stainless steel consisting of ferrite and austenite phases, and its chemical composition is shown in Table 1. The powder morphology is shown in Figure 1. It can be seen that the raw powder showed spherical morphology and a smooth surface, and obvious cellular structures and grain boundaries could be clearly observed in Figure 1b.

**Figure 2:**
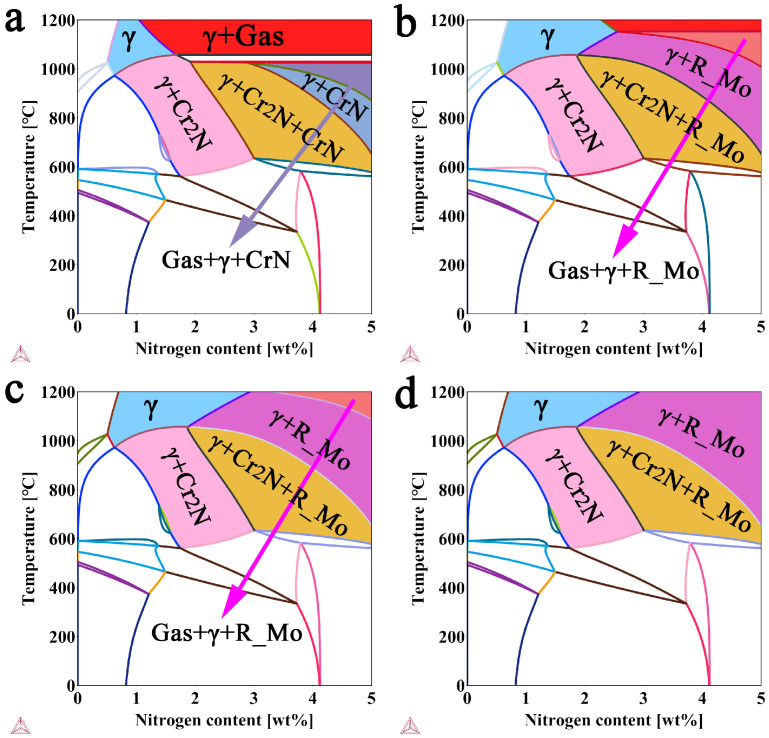
Thermodynamic equilibrium phase diagram of FeCr17Mn12Mo3.5N steel at different pressures: (**a**) 0.1 MPa; (**b**) 1.0 MPa; (**c**) 3.0 MPa; (**d**) 5.0 MPa.

As we all know, the pressure is a crucial process parameter that influences the phase and microstructure and nitriding efficiency, thus the thermodynamic equilibrium phase diagrams of FeCr17Mn12Mo3.5N steel at 0.1, 1.0, 3.0 and 5.0 MPa were calculated using Thermo-Calc 2020 software with the TCFE 8 thermodynamic database, as depicted in Figure 2. The results reveal that the γ-austenite phase region gradually expands with increasing pressure, confirming the rationality of the high-pressure solid powder nitriding process. Furthermore, the observed progressive contraction of the gas + γ-austenite region with increasing pressure demonstrates that both the phase constituents and the nitrogen content are strongly pressure-dependent. Accordingly, it can be inferred that high pressure can achieve an efficient synergy between “nitrogen increasing” and “nitrogen controlling”. However, the area increase in the area of γ-austenite phase is slows when the pressure exceeds 3 MPa, as shown in Figure 2c. Therefore, a nitriding pressure not exceeding 3 MPa was adopted.

Based on the above analysss, the HNASS powders were prepared using the following procedure. The raw FeCr17Mn12Mo3.5N powders were evenly spread in a quartz crucible and then put into a high-pressure tube furnace for nitriding treatments. The nitriding treatment conditions were set as follows: pressures of 0.5, 1.0, 1.5, 2.0, 2.5, and 3.0 MPa; temperatures of 800, 850, 900, 950, and 1000 °C; and durations of 0.5, 1.0, 1.5, 2.0, and 2.5 h, respectively. The specific process parameters are shown in Table 2. After the nitriding treatment, the furnace was cooled naturally to room temperature in N_2_ atmosphere. It is particularly noteworthy that effectively inhibiting the formation of oxide layers on the powder surface is critical for promoting the nitriding reaction. Therefore, high-purity nitrogen (99.999 wt.%) was selected for the experiment, and the furnace was subjected to multiple purging and vacuuming treatments. Importantly, the copper treatment was also carried out to reduce the free oxygen in the nitriding chamber, which can effectively restrain the occurrence of oxidation reactions on the powder surface.

### 2.2. Material Characterizations

The oxygen and nitrogen contents were analyzed using an oxygen, nitrogen and hydrogen analyzer (ONH, ONH836, LECO, (St. Joseph, MI, USA) and each measurement was repeated five times to obtain an average value. The phase composition was characterized by an X-ray diffraction (XRD, D/MAX2500PC, RIGAKU, Tokyo, Japan) using Cu-Kα radiation from 25° to 85° with a step size of 0.24°. The surface and cross-section morphologies of the powder were observed using a scanning electron microscope (SEM, ZEISS SUPRATM55, ZEISS, Oberkochen, Germany). The elemental composition and distribution of the powder samples were analyzed using an electron probe microanalyzer (EPMA, JXA-8230, JEOL, Tokyo, Japan). The microstructure was further characterized by transmission electron microscopy (STEM-EDS, JEM-F200, JEOL, Tokyo, Japan). The TEM samples were prepared using the microscopic processing system with a focus ion beam (FIB). The surface chemical characteristics were explored using X-ray photoelectron spectroscopy (XPS, AXISULTRA DLD, KRATOS, Manchester, UK).

## 3. Results and Discussion

### 3.1. Microstructure Characterization

**Figure 3:**
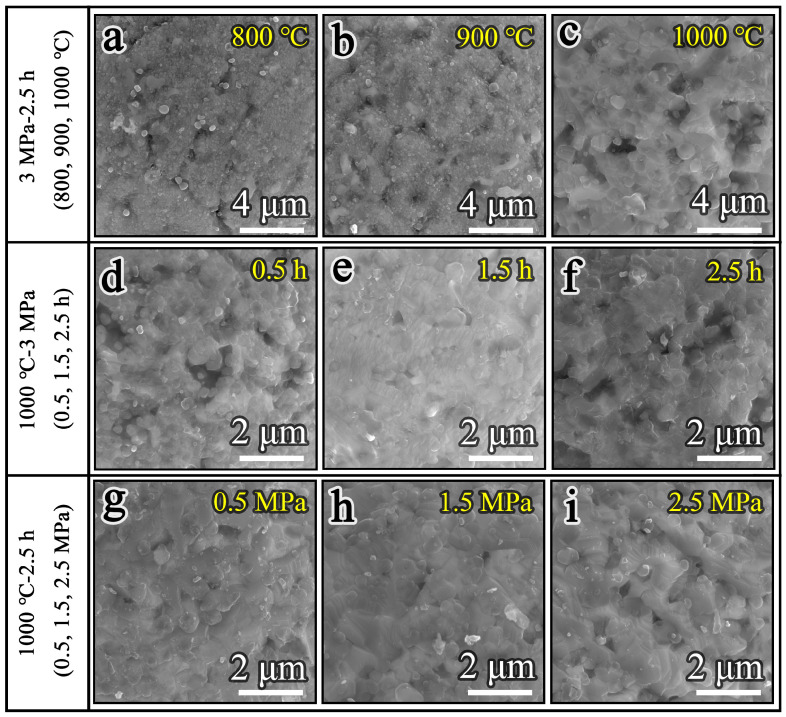
SEM images of the nitrided powders: (**a**) 800 °C; (**b**) 900 °C; (**c**) 1000 °C; (**d**) 0.5 h; (**e**) 1.5 h; (**f**) 2.5 h; (**g**) 0.5 MPa; (**h**) 1.5 MPa; (**i**) 2.5 MPa.

**Figure 4:**
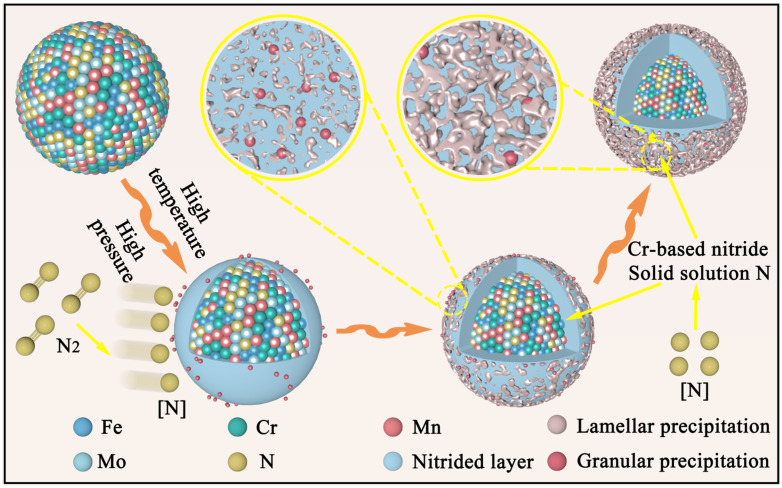
Evolution mechanism of powder surface morphology.

The surface morphology of the nitrided powders prepared under different nitriding parameters is shown in Figure 3. As observed, the nitrided powders retain a structurally intact matrix with no evidence of cracking, indicating their high stability under the processing conditions. The effect of nitriding temperature on the morphology of the powders is investigated first, as shown in Figure 3a–c. Compared with the raw powder, the nitrided powder exhibits a rougher surface morphology, with the cellular structures being largely obscured by numerous granular precipitates covering the surface (Figure 3a). Moreover, as the temperature increases, both the number and size of the granular precipitates increase gradually. When the temperature is 900 °C, irregular lamellar precipitates appear on the surface of the powder. As the temperature is further increased to 1000 °C, the original cellular structures on the powder surface become completely covered by granular and lamellar precipitates, as shown in Figure 3c. Figure 3d–f illustrate the evolution of the powder surface morphology with increasing nitriding times of 0.5, 1.5 and 2.5 h, respectively. It can be seen that the morphology of the powder surface changes significantly with increasing nitriding time. The initial fine granular precipitates gradually grow and finally transform into lamellar precipitates. A comparison reveals that the surface morphology shows no significant differences under varying nitriding pressures, consistently featuring a mixed structure of numerous lamellar precipitates and a small number of granular precipitates, as shown in Figure 3g–i. In view of the above results, it can be concluded that the influence of nitriding temperature on the surface morphology is greater than that of time and pressure. The microscopic morphology of the powder surface follows an evolution sequences of granular precipitates, particle growth, and lamellar precipitates during the nitriding process of the powder, as shown in Figure 4. The proposed nitriding mechanism is based on the observations in Figure 3.

**Figure 5:**
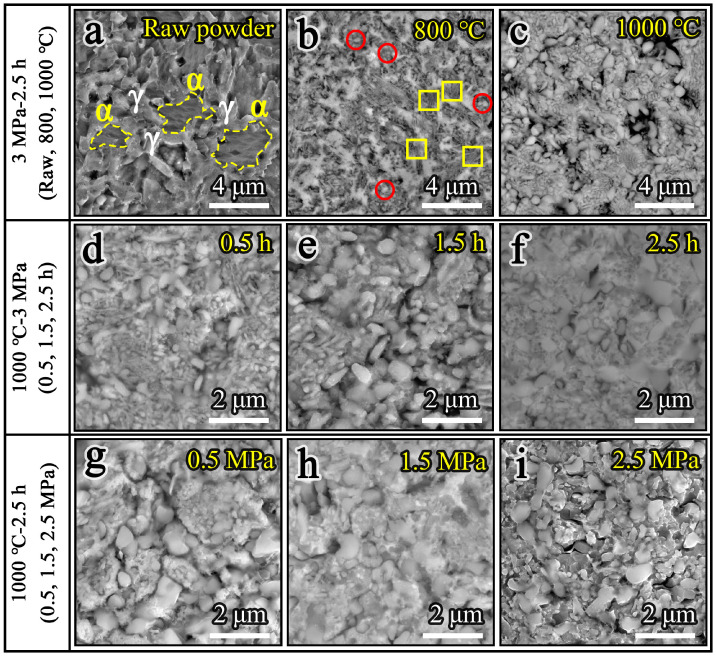
SEM images of the cross-section of the raw and nitrided powders: (**a**) raw powder; (**b**) 800 °C; (**c**) 1000 °C; (**d**) 0.5 h; (**e**) 1.5 h; (**f**) 2.5 h; (**g**) 0.5 MPa; (**h**) 1.5 MPa; (**i**) 2.5 MPa.

To investigate the evolution of the cross-sectional morphology and precipitates in the powders after nitriding, both the raw and nitrided powders were mounted in a conductive thermosetting resin on the surface of the mold. Then the prepared samples were successively polished using sandpapers with different grit sizes and etched. Etching was performed by immersion for 10 s in an etching a solution consisting of 1 g CuSO_4_ + 5 mL HCl + 5 mL H_2_O. The cross-sectional morphologies of the etched raw powder and nitrided powders are shown in Figure 5. Because the corrosion resistance of the austenite phase is superior to that of the ferrite phase, the protrusions are identified as the austenite phase. As seen in Figure 5a, the cross-section of the raw powder exhibits a mixed microstructure of austenite and ferrite. After nitriding treatment at 800 °C and 1000 °C, the microstructures of the cross-sections of the nitrided powders are shown in Figure 5b,c. At 800 °C, the cross-section of the nitrided powder presents fine granular (indicated by circles) and flocculent lamellar precipitates (indicated by squares). With the increase in nitriding temperature, the granular precipitates obviously grew, while the flocculent lamellar precipitates disappeared, as shown in Figure 5c. This indicates that the nitriding temperature plays a critical role in determining the cross-sectional microstructure of the powder. To further investigate the effect of nitriding time on the microstructure of the powder, the cross-sectional morphologies of the powders with nitriding times of 0.5, 1.5 and 2.5 h were characterized as shown in Figure 5e,f. With the increase in nitriding time, the granular precipitates in the powder matrix gradually grew and became larger, indicating that an increase in nitriding time leads to the further growth of precipitates. This shows that nitriding time is an important factor affecting the size of the precipitates. Figure 5g–i show the microstructure of the cross-section of the nitrided powders under different nitriding pressures. It can be observed that with the increase in nitriding pressure, the size of the precipitates shows a decreasing trend, which may be attributed to the high diffusion flux of nitrogen. The abundant active nitrogen atoms combined with Cr atoms in the matrix accelerate the generation of nitrides, which leads to an increase in the number of precipitates and a decrease in their size. This phenomenon is due to the fact that according to Sieverts’ law the equilibrium nitrogen potential at the powder surface is proportional to the square root of the nitrogen partial pressure. This causes a huge concentration gradient and leads to a sharp increase in the diffusion flux of active nitrogen atoms into the interior, which further reduces the diffusion time required for nitrogen atoms to reach the same depth. Ultimately, efficient nitriding can be achieved with a shortened nitriding time, while the growth of precipitates is effectively controlled [4,35,36].

### 3.2. Nitrogen Content and Phase Composition Analysis

**Figure 6:**
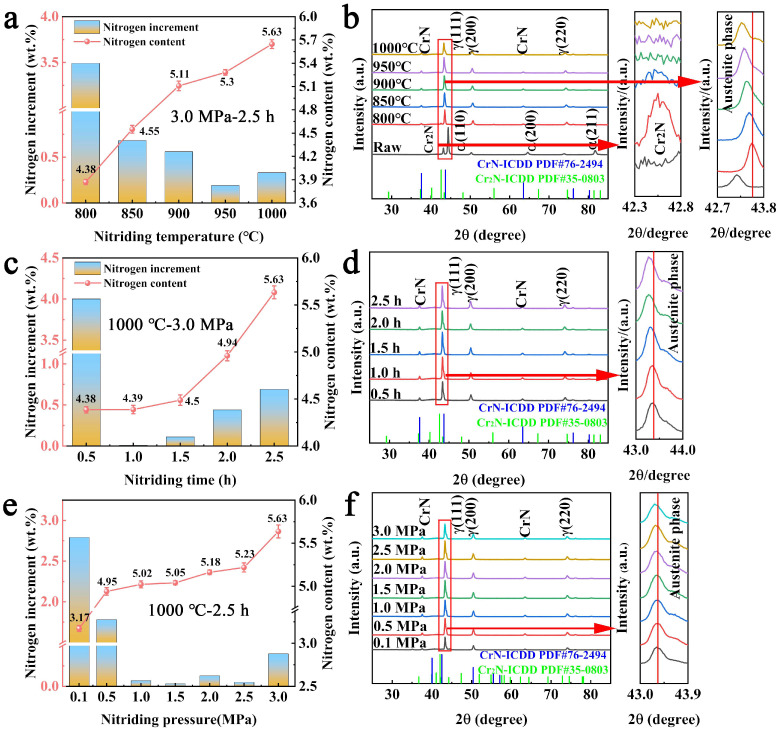
Nitrogen content and XRD patterns of powder under different nitriding processes: (**a**,**b**) temperature; (**c**,**d**) time; (**e**,**f**) pressure.

The nitrogen content and phase composition of the raw powder and nitrided powders are shown in Figure 6. The nitrogen content of the raw powder is determined to be 0.38 wt.%. After high-pressure nitriding, the nitrogen content increases significantly. For the nitrided powders obtained at different nitriding temperatures, as shown in Figure 6a, the nitrogen content increases with temperature, reaching up to 5.63 wt.%. Compared with s previous report where the HNASS powders with a nitrogen content of only of 1.33 wt.% were obtained by N2 nitriding for 1 h at atmospheric pressure [29], the nitrogen content (4.38~5.63 wt.%) of powders prepared under a high-pressure atmosphere in this work is higher. The corresponding XRD analysis reveals that the raw powder is mainly composed of the δ-ferrite phase with a small amount of γ-austenite, as shown in Figure 6b. After high-pressure nitriding, the δ-ferrite phase disappears and the phase transforms to γ-austenite with the appearance of a certain number of nitrides due to the increase in nitrogen content; this indicates that the ultra-HNASS powder can be successfully synthesized [37,38]. In addition, as the nitriding temperature increases, the diffraction peaks of the γ-austenite phase gradually shift to lower angles due to lattice expansion caused by the continuous increase in the solid solution nitrogen content [39,40]. It should be noted that the CrN phase occurs in all nitrided powders, while the Cr_2_N phase is only observed in the nitride powder obtained at temperatures not exceeding 850 °C. This is because the formation of Cr_2_N is related to the concentration of active nitrogen atoms in the powder. When the active nitrogen atom concentration is low, Cr elements with a high affinity for nitrogen will preferentially generate Cr_2_N, which transitions to CrN as the nitrogen concentration continues to increase. This phenomenon has been reported in previous research, which suggests that the transformation mechanism of Cr-based nitrides follows a sequence of Cr_2_N + CrN, CrN with increasing nitrogen content [41,42]. This transformation explains the aforementioned microstructural changes shown in Figure 5b,c, where the flocculent and lamellar precipitates (assigned to Cr_2_N) transform into granular precipitates (assigned to CrN) with increasing nitrogen content.

The nitrogen content of the powder at various nitriding times is presented in Figure 6c. The results show that the nitrogen content increases significantly with an increase in nitriding time. When the nitriding time is 0.5 h, 1.0 h, 1.5 h, 2.0 h and 2.5 h, the nitrogen content is 4.38, 4.39, 4.5, 4.94 and 5.63 wt.%, respectively. It is worth noting that the increase of nitrogen content in the powder at different times shows certain differences compared with the influence of nitriding temperature. With the increase in nitriding time, the rate of nitrogen enrichment continuously increases, and the increment becomes particularly obvious when the nitriding time reaches 2.0 h and 2.5 h. Figure 6d shows the XRD patterns of the powders at different nitriding times. It can be clearly observed that the peaks positions of the γ-austenite phase show significant shifts at nitriding times of 2.0 h and 2.5 h. Combined with the SEM morphology analysis of Figure 5d–f, it can be inferred that the significant increase in nitrogen content is mainly attributed to the synergistic mechanism of the increase in the size of nitrided precipitates and the amount of nitrogen in the form of a solid solution in the powder. Furthermore, the nitrogen content of the powder at 1000 °C for 2.5 h under different nitriding pressures is shown in Figure 6e. The results show that the nitrogen content slightly increases with an increase in nitriding pressure, reaching 3.17, 4.95, 5.02, 5.05, 5.18, 5.23, and 5.63 wt.% for pressures of 0.1 MPa, 0.5 MPa, 1.0 MPa, 1.5 MPa, 2.0 MPa, 2.5 MPa, and 3.0 MPa, respectively. It is worth noting that the nitrided powder with a nitrogen content of 3.17 wt.% obtained under 0.1 Mpa has a nitrogen that content is much lower than that of the nitrided powders under a high-pressure atmosphere, thereby verifying the positive effect of high pressure on the nitriding process. The XRD patterns in Figure 6f show that the γ-austenite diffraction peaks remain essentially unchanged with increasing nitrogen content, indicating that the slight increase is primarily attributable to nitride formation rather than lattice expansion from the solid solution. The above-mentioned insignificant shift in the diffraction peaks is attributed to the limited increase in solution nitrogen content. This significant finding provides valuable guidance for the tuning of nanoscale nitrides in the powder.

### 3.3. TEM Characterization

**Figure 7:**
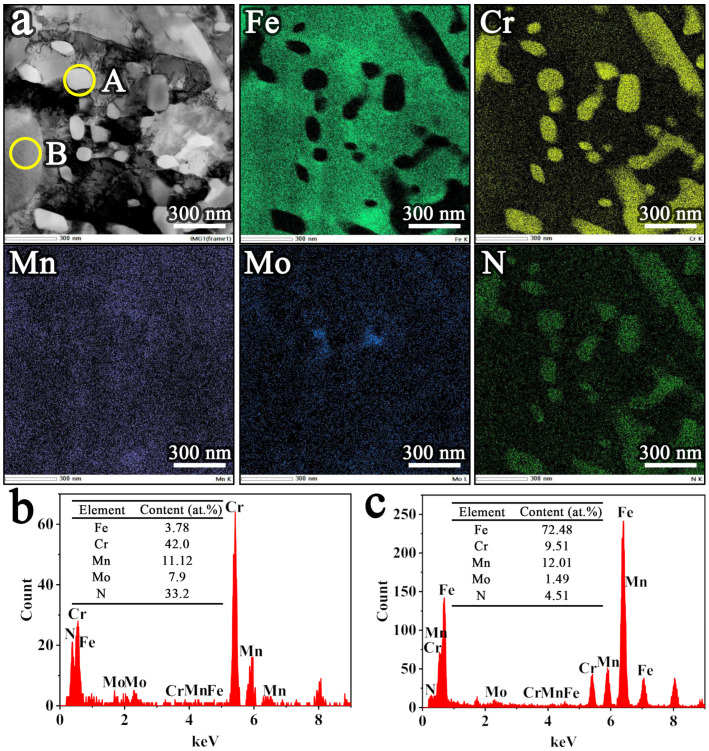
STEM-EDS characterization of nitrided powder: (**a**) TEM image and elemental distribution; (**b**) EDS spectra from the A region; (**c**) EDS spectra from the B region.

The microstructure of nitrided powders with a nitrogen content of 4.55 wt.% was further observed by TEM, as shown in Figure 7a. Figure 7a shows the microstructure and elemental distribution. In the A region, a distinct enrichment behavior of Cr and N elements is evident, further confirming that the granular precipitates are Cr-based nitrides. Furthermore, it is observed that the granular precipitates are nanosized, indicating that a synergistic effect of grain refinement and precipitation strengthening is achieved. No element enrichment phenomenon is observed in B region, which indicates that all elements are uniformly distributed in this region. Combined with the XRD results, the powder matrix is identified as the austenite phase. Moreover, a STEM-EDS analysis was further performed to identify and verify the composition of the powder matrix and the Cr-based nitrides, as shown in Figure 7b,c. The results indicate that the Cr: N atomic ratio of the granular precipitate is close to 1:1, confirming the formation of CrN precipitates. Also, the fact that the matrix is a N-enriched austenite phase is further confirmed.

To further understand the sizes of the austenite grains and nitrides in the bulk powder, the Williamson—Hall formula and a statistical method were used to analyze the dimensions of the two phases, respectively. Specifically, the austenite grain sizes at different temperatures were calculated, and the particle size distribution of nitrides in the powder with a nitrogen content of 4.55 wt.% was statistically analyzed. The grain size was estimated based on the XRD patterns using the Williamson—Hall equation [31]:

$$\beta \cos\theta =0.89\frac{\lambda }{d}+2\varepsilon \sin\theta$$

where *β* is the full width at half maximum of the diffraction peaks, *θ* is the Bragg’s diffraction angle, *λ* is the wavelength of the X-rays, *d* is the grain size and ε is the internal lattice strain.

**Figure 8:**
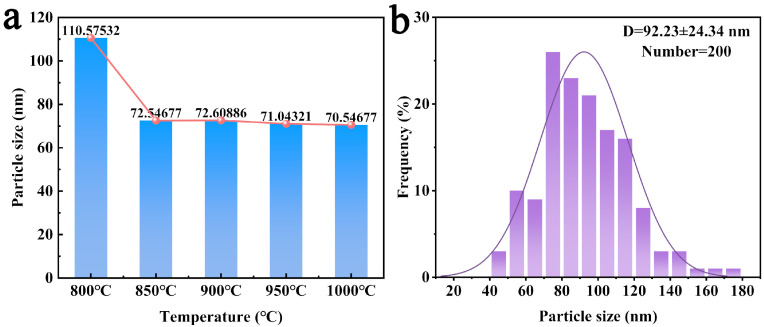
Particle size statistics (**a**) austenite; (**b**) CrN.

Figure 8 shows the sizes of the austenite grains and nitrides. The results indicate that the austenite grain size is at the nanometer scale and decreases gradually with increasing temperature. The statistical results of the nitride particle size show that the average particle size is 92.23 ± 24.34 nm.

**Figure 9:**
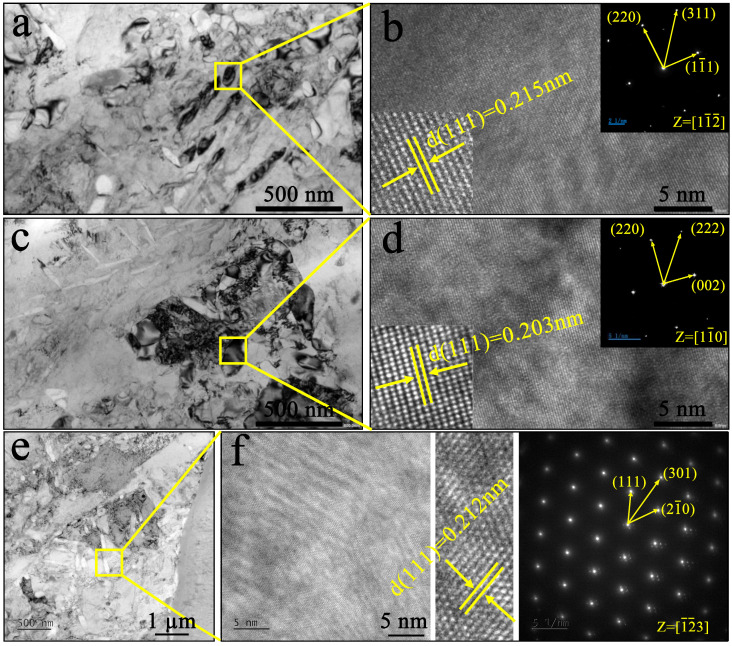
TEM images of nitrided powder. (**a**) TEM image of the sample slice; (**b**) HRTEM and SAED of Figure (**a**); (**c**) TEM image of the sample slice; (**d**) HRTEM and SAED of figure (**c**); (**e**) TEM image of the sample slice; (**f**) HRTEM and SAED of Figure (**e**).

Figure 9 shows the TEM images. Before analysis, the lattice spacings were calibrated using an external Si standard (a = 0.5431 nm). Figure 9a–e show that the grain boundaries can be clearly observed. The HRTEM images in Figure 9b–f show clear lattice fringes. From the SAED patterns in the inset of Figure 9b, the phase displays an FCC structure, which is assigned to the austenite phase. In addition, the lattice spacing is measured to be 0.215 nm, which is ascribed to the enlarged (111) crystal face spacing due to the occupation of interstitial sites in the FCC structure of austenite by a large amount of nitrogen, which causes lattice expansion, consistent with the nitrogen content and XRD analysis. The SAED pattern in Figure 9d indicates that the phase is CrN, and the interplanar spacing of the CrN (111) plane is 0.203 nm. As shown in Figure 9e,f, HRTEM and SAED analyses show that the precipitates exhibit an HCP structure with a lattice spacing of 0.212 nm, which corresponds well to hexagonal Cr_2_N. These TEM observations provide direct evidence for the formation of Cr_2_N at this temperature. Combined with the XRD results, they confirm the proposed evolution of nitride phases from Cr_2_N to CrN as the nitriding temperature increases. Meanwhile, the sizes of the austenite grains and CrN precipitates observed in the TEM bright field are at the nanoscale, indicating that the powder has excellent physical and chemical properties, as shown in Figure 9a,c.

### 3.4. EPMA Characterization

**Figure 10:**
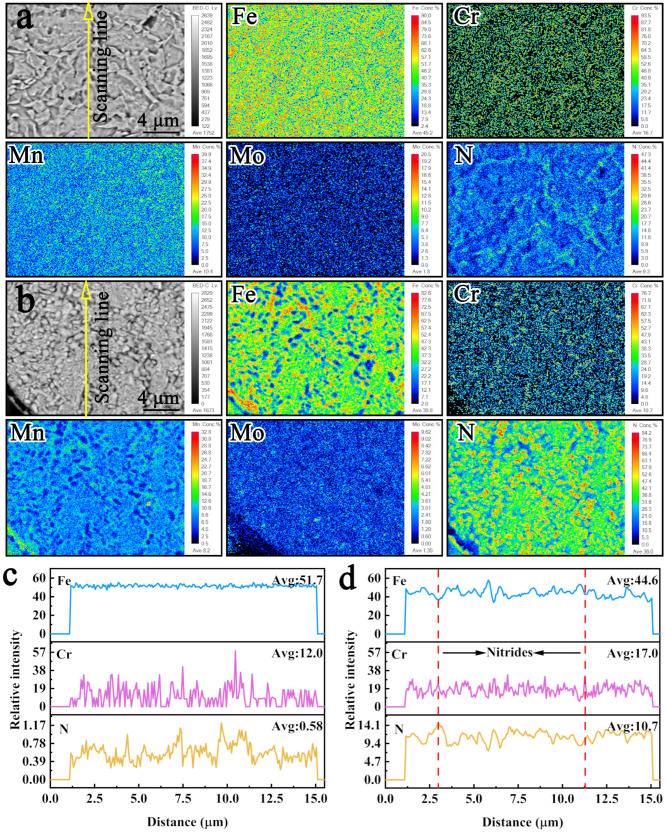
EPMA images of powder: (**a**) raw powder and (**b**) nitrided powder; EPMA line scans of powder: (**c**) raw powder and (**d**) nitrided powder.

To further analyze the nitriding behavior and the chemical state of nitrogen in the powder, EPMA characterization was performed on the cross-section of the raw powder and the nitrided powder with a nitrogen content of 5.63 wt.%, as shown in Figure 10. Figure 10a shows that the elements are uniformly distributed in the raw powder matrix. Only the N elements exhibits a slight element enrichment phenomenon at the protrusions, while other elements do not show this phenomenon, indicating that the enriched N areas correspond to the austenite phase. This result is consistent with the SEM result in Figure 5a. Figure 10b shows the element distribution of the cross-section of the nitrided powder. Compared with the raw powder, the distribution of Fe elements in the cross-section of the powder after high-pressure nitriding is significantly non-uniform, with numerous Fe-depleted zones corresponding to the granular precipitation regions. Combined with the elemental distribution of other elements, it is found that the Fe-depleted sites show a significant enrichment of phenomenon of Cr and N elements, indicating that a certain number of nitrides precipitated after high-pressure nitriding. Figure 10c,d demonstrate the line scan results of the raw powder and nitrided powder, respectively. After nitriding, significant changes in the quantitative intensity of Fe, Cr and N were observed in the powder. In the nitrided powder, regions with high Cr signal intensity exhibit correspondingly high N intensity, along with reduced Fe intensity. This indicates that the granular precipitates shown in the figure are chromium nitrides. Meanwhile, the signal intensity of N exhibits no declining trend with increasing line-scan depth, demonstrating a uniform nitriding effect in the powder. From the view of the whole powder matrix, the solid solution nitrogen is uniformly distributed and nanoscale nitrides are dispersed as precipitates in the powder matrix. This indicates that the high-pressure nitriding process is suitable and feasible for the rapid homogeneous nitriding of solid powders.

### 3.5. XPS Characterization

**Figure 11:**
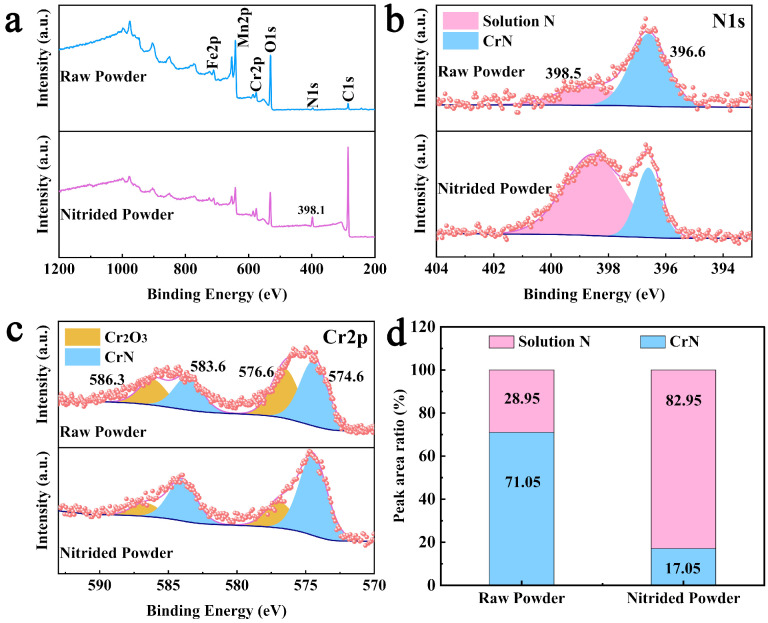
The raw and nitrided powder XPS spectra: (**a**) total spectra; (**b**) N1s; (**c**) Cr2p; (**d**) the nitrogen content.

To further investigate the chemical state and ratio of nitrogen, XPS analysis was conducted on the raw powder and nitrided powder with a nitrogen content of 5.63 wt.%, as shown in Figure 11. To minimize the influence of surface contamination and any native oxide layer, the powder samples were subjected to Ar^+^ ion sputtering to a depth of 10 nm before XPS analysis. And all spectra were charge-corrected to adventitious C 1 s at 284.8 eV. The specific fitting parameters are shown in Table 3. From the survey spectra in Figure 11a, it can be seen that a new peak with a binding energy of 398.1 eV corresponding to N1s appears in the nitrided powders, indicating that the content of nitrogen increases after high-pressure nitriding. In Figure 11b, the N1s spectra show that the peaks located at 396.6 eV and 398.5 eV are assigned to CrN and solid solution N, respectively. By comparison, the peak of the solid solution N becomes obviously stronger after high-pressure nitriding, indicating that a large amount of nitrogen was introduced in the form of a solid solution [43,44], which can improve the stability of the austenite phase and the properties of the powder [45]. The spectra of Cr2p are shown in Figure 11c. The fitted peaks at 574.6/583.6 eV and 576.6/586.3 eV can be indexed to CrN and Cr_2_O_3_, respectively [46]. Based on the XPS analysis, the ratio of the N1s peak area corresponding to solid-solution nitrogen increased from ~28.95% in the raw powder to ~82.95% in the nitrided powder, indicating that high-pressure nitriding effectively introduces nitrogen into the solid solution within the surface layer, as shown in Figure 11d.

**Figure 12:**
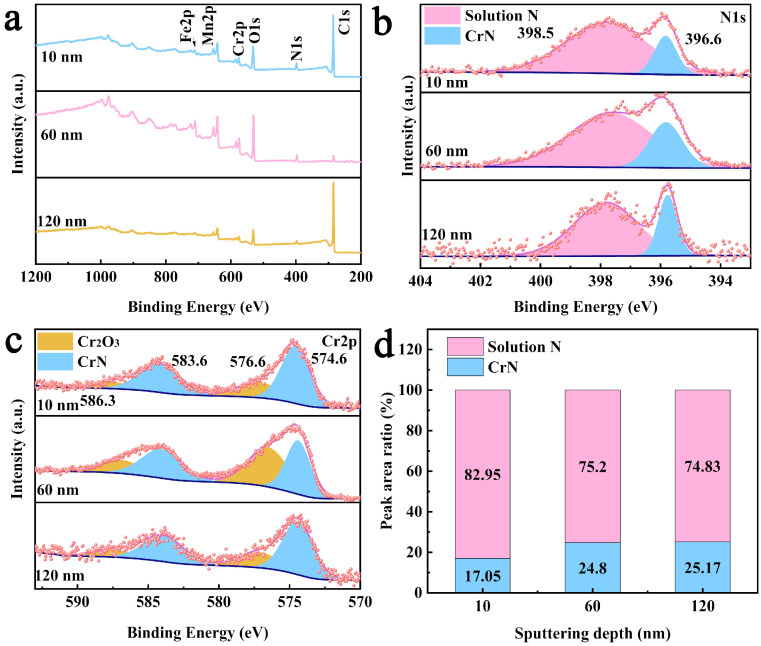
The nitrided powder XPS spectra: (**a**) total spectra; (**b**) N1s; (**c**) Cr2p; (**d**) the peak area ratio of nitrogen in different chemical states.

To further explore the chemical state and ratio of nitrogen in the interior of the powder, XPS characterization was conducted on the nitrided powder with a nitrogen content of 5.63 wt.%, as shown in Figure 12. Before analysis, the sample was sputtered with Ar^+^ ions to depths of 10 nm, 60 nm, and 120 nm within the powder. The specific fitting parameters are shown in Table 3. From the survey spectra in Figure 12a, it can be seen that the intensity of the N1s peak shows no significant variation, indicating that nitrogen is distributed relatively uniformly within the nitrided powder. By comparing the fine spectra of the N1s, it was found that the ratio of the different elemental states did not change significantly at different depths, indicating that the distribution of nitrogen within the powder was relatively uniform, as shown in Figure 12b. To precisely quantify the ratios of chemical states for nitrogen, peak fitting of the N1s spectrum was performed, and the results are shown in Figure 12d. After sputtering to a depth of 10 nm (corresponding to the powder surface layer), the peak area ratios of solid solution nitrogen to nitrides were ~82.95% and ~17.05%, respectively. After sputtering to a depth of 60 nm, the peak area ratios were ~75.2% and ~24.8%, and after sputtering to a depth of 120 nm, they became ~74.83% and ~25.17%. These results indicate that while the surface layer has slightly higher solid solution nitrogen, the middle and inner regions exhibit a consistent chemical state ratio. In summary, the distribution of solid solution nitrogen and nitrides within the powder is relatively uniform.

### 3.6. Nitriding Mechanism

**Figure 13:**
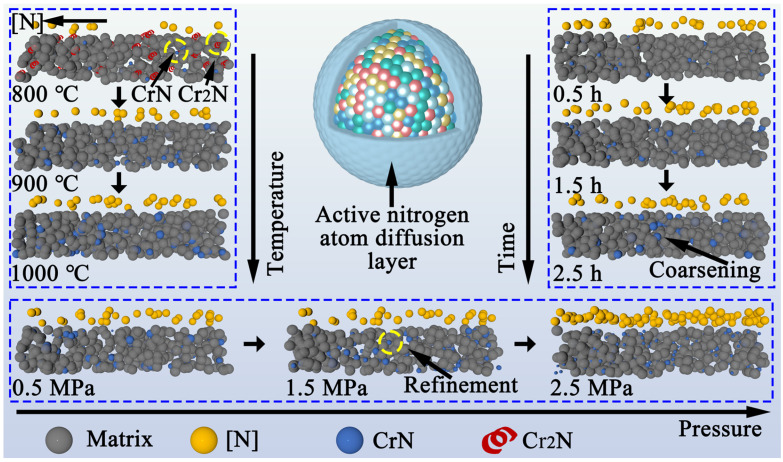
Diagram of the nitriding mechanism of the powder.

Figure 13 shows the nitriding mechanism affected by different process parameters. During the nitriding process, active nitrogen atoms are first adsorbed on the powder surface to form a diffusion layer. In terms of temperature, at 800 °C, the number of active nitrogen atoms is relatively low, and Cr atoms with high nitrogen affinity combine with active nitrogen atoms to preferentially generate Cr_2_N. As the temperature rises and thermodynamic conditions improve, the efficiency of nitrogen gas decomposition into active nitrogen atoms accelerates, and the number of active nitrogen atoms on the powder surface increases. As a result, the Cr atoms react with the active nitrogen atoms to form CrN. This is because Cr_2_N is unstable under high nitrogen potential conditions and undergoes a further reaction with active nitrogen atoms to transform into the more stable CrN. This results in a nitride evolution from the coexistence of Cr_2_N and CrN to a single CrN phase, accompanied by the coarsening of the CrN precipitates. In terms of time, an increase in nitriding time does not affect the change of nitrogen potential on the powder surface. It primarily influences the diffusion depth of active nitrogen atoms and internal reactions. Thus, with an increase in nitriding time, the continuous diffusion of active nitrogen atoms leads to the coarsening of CrN precipitates. As we all know, pressure is an important factor for optimizing the size and distribution of nitrides. The increase in pressure accelerates the rate of nitrogen gas conversion into active nitrogen atoms, which leads to a rapid rise in the nitrogen potential on the powder surface. High nitrogen potential not only increases the diffusion flux of active nitrogen atoms, but more importantly creates a relatively high nitrogen saturation within the powder. According to classical nucleation theory, high supersaturation of active nitrogen atoms increases the nucleation rate, leading to the formation of more CrN crystal nuclei [47]. The rapid and extensive formation of CrN crystal nuclei leads to a decrease in their size, making their distribution more uniform in the powder matrix. In conclusion, the nitrogen content, nitride size and distribution in the powder can be precisely controlled by the synergetic regulation of “temperature–time–pressure” parameters.

## 4. Conclusions

In this study, ultra-HNASS powders with different nitrogen contents were obtained by an innovative high-pressure nitriding process of solid powders. The HNASS powder achieved an ultra-high nitrogen content of up to 5.63 wt.% while maintaining a single austenite phase. It was found that the phase composition of the powder can be tuned by temperature. As the temperature increases, the ferrite phase gradually transforms into the austenite phase. Meanwhile, the nitrides also evolve from Cr_2_N + CrN to CrN. TEM analysis indicates that the size of the austenite grains and the nitrides in the powder are at the nanometer level, and the nitrides are dispersed as precipitates in the matrix. Moreover, the high-pressure nitriding process can optimize the chemical state of nitrogen in the powder. Based on XPS analysis of the powder surface, the peak area ratios of solid-solution nitrogen and nitrides in the N1s spectrum were calculated to be ~82.95% and ~17.05%, respectively. The above research indicates that the high-pressure nitriding process plays a positive role in the size and distribution of nitride precipitates. In summary, the size and distribution of nitrides and the nitrogen content strongly depend on the process parameters of temperature, time and pressure, and they can be effectively regulated by process parameters, which indicates that this strategy is effective and efficient for the controllable preparation of HNASS.

However, at the highest N content (5.63 wt.%), a small fraction of CrN precipitates (~17.05% by XPS) is unavoidable. Further optimization may target even finer precipitate dispersion. Compared to conventional gas atomization at 0.1 MPa, the high-pressure process increases the N content by a factor of ~15, but requires specialized equipment. The trade-off between performance and cost should be evaluated for industrial applications.

## Figures and Tables

**Table 1:** The chemical composition of FeCr17Mn12Mo3.5N raw powders.

Element	Fe	Cr	Mn	Mo	N	O	Si	C
Content (wt.%)	Bal.	17.24	12.11	3.56	0.38	0.052	0.34	0.014

**Table 2:** The nitriding process parameters.

Sample ID	Temperature (°C)	Time (h)	Pressure (MPa)	Nitrogen Content (wt.%)
A1	800	2.5	3.0	4.38
A2	850	2.5	3.0	4.55
A3	900	2.5	3.0	5.11
A4	950	2.5	3.0	5.3
A5	1000	2.5	3.0	5.63
B1	1000	0.5	3.0	4.38
B2	1000	1.0	3.0	4.39
B3	1000	1.5	3.0	4.5
B4	1000	2.0	3.0	4.94
B5	1000	2.5	3.0	5.63
C1	1000	2.5	0.5	4.95
C2	1000	2.5	1.0	5.02
C3	1000	2.5	1.5	5.05
C4	1000	2.5	2.0	5.18
C5	1000	2.5	2.5	5.23
C6	1000	2.5	3.0	5.63

**Table 3:** The XPS fitting parameters.

Sample	Chemical State	Binding Energies (eV)	FWHM (eV)	Peak Shape	Background Type	Software
Raw Powder—10 nm	CrN	396.63	1.54	GL (30)	Shirley	Thermo Avantage v5.948
	Solution N	398.56	2.43			
	Cr_2_O_3_	576.63	2.39			
		586.28	2.40			
	CrN	574.64	2.33			
		583.59	2.37			
Nitrides Powder—10 nm	CrN	396.56	0.85	GL (30)	Shirley	Thermo Avantage v5.948
	Solution N	398.43	2.91			
	Cr_2_O_3_	576.63	2.35			
		586.33	2.37			
	CrN	574.6	2.42			
		583.62	2.33			
Nitrides Powder—60 nm	CrN	396.55	0.91	GL (30)	Shirley	Thermo Avantage v5.948
	Solution N	398.45	2.40			
	Cr_2_O_3_	576.64	2.42			
		586.28	2.38			
	CrN	574.55	2.39			
		583.67	2.38			
Nitrides Powder—120 nm	CrN	396.56	0.91	GL (30)	Shirley	Thermo Avantage v5.948
	Solution N	398.68	2.88			
	Cr_2_O_3_	576.65	2.41			
		586.33	2.38			
	CrN	574.66	2.38			
		583.63	2.43			

## Data Availability

The original contributions presented in this study are included in the article. Further inquiries can be directed to the corresponding authors.
